# Early B-cell Factor 3–Related Genetic Disease Can Mimic Urofacial Syndrome

**DOI:** 10.1016/j.ekir.2020.07.001

**Published:** 2020-07-14

**Authors:** J. Robert Harkness, Glenda M. Beaman, Keng W. Teik, Sangeet Sidhu, John A. Sayer, Heather J. Cordell, Huw B. Thomas, Katherine Wood, Helen M. Stuart, Adrian S. Woolf, William G. Newman

**Affiliations:** 1 Manchester Centre for Genomic Medicine, Manchester University NHS Foundation Trust, Health Innovation Manchester, Manchester, UK; 2 Division of Evolution and Genomic Sciences, Faculty of Biology, Medicine and Health Sciences, University of Manchester, Manchester, UK; 3 Genetic Department, Hospital Kuala Lumpur, Kuala Lumpur, Malaysia; 4 Paediatric Department, Hospital Pulau Pinang, Pulau Pinang, Malaysia; 5 Clinical Medicine Institute, Faculty of Medical Sciences, Newcastle University, Newcastle Upon Tyne, UK; 6 Renal Services, The Newcastle upon Tyne Hospitals NHS Foundation Trust, Newcastle upon Tyne, UK; 7 NIHR Newcastle Biomedical Research Centre, Newcastle University, Newcastle upon Tyne, UK; 8 Population Health Sciences Institute, Faculty of Medical Sciences, Newcastle University, Newcastle upon Tyne, UK; 9 Division of Cell Matrix Biology and Regenerative Medicine, School of Biological Sciences, Faculty of Biology Medicine and Health, University of Manchester, Manchester, UK; 10 Royal Manchester Children's Hospital, Manchester University NHS Foundation Trust, Manchester Academic Health Science Centre, Manchester, UK

## Introduction

Several decades ago, Bernardo Ochoa^1^ described a rare but potentially devastating inherited disease that is now called urofacial, or Ochoa, syndrome (UFS).^2^ UFS is characterized by 2 features. First, a so-called “non-neurogenic neurogenic” dyssynergic bladder in which functional bladder outflow obstruction causes incomplete voiding, vesicoureteric reflux (VUR), ascending urosepsis, pyelonephritis, and renal failure. Second, a grimace where the corners of the mouth become downturned on smiling, so that the face appears to be crying. Severe constipation is reported in approximately two-thirds of cases.^1^ Ochoa’s^1^ cohort was from Colombia, but UFS has subsequently been reported worldwide and cases totaled at least 150.^2^ UFS is an autosomal recessive disorder and most cases are caused by biallelic pathogenic variants in either of 2 genes: *HPSE2* (Mendelian Inheritance in Man (MIM) 613469)^3^, ^4^, ^5^ encoding heparanase-2, which inhibits the enzymatic activity of classical heparanase, or *LRIG2* (MIM 615112)^6^ encoding leucine-rich repeats and Ig-like domains, a protein that may modulate growth factor signaling. Heparanase 2 and LRIG2 proteins are present in pelvic ganglia and in bladder autonomic nerves emanating from these ganglia,^5^, ^6^, ^7^ and the patterns of bladder nerves are abnormal in mice carrying biallelic variants in either *Hpse2* or *Lrig2.*^5^, ^6^, ^7^ Thus, UFS features a peripheral neuropathy affecting the bladder, whereas the cause of the grimace requires further study.

A minority of people with an apparent clinical diagnosis of UFS do not carry variants in *HPSE2* or *LRIG2*.^5^, ^6^, ^7^ The current report draws attention to a different genetic syndrome, which can mimic UFS and be associated with renal failure. We describe one such individual with a heterozygous missense predicted pathogenic variant in *early B-cell factor 3* (*EBF3*)*,* a gene encoding a transcription factor and associated with hypotonia, ataxia, and developmental delay syndrome (HADDS; MIM 617330).^8^^,^^9^^,^S1 We proceeded to seek variants in *EBF3* in a UK cohort with familial primary nonsyndromic VURS2 and reviewed the published literature of HADDS to determine whether other such individuals had renal tract disease.

## Case Presentation

**Figure 1:**
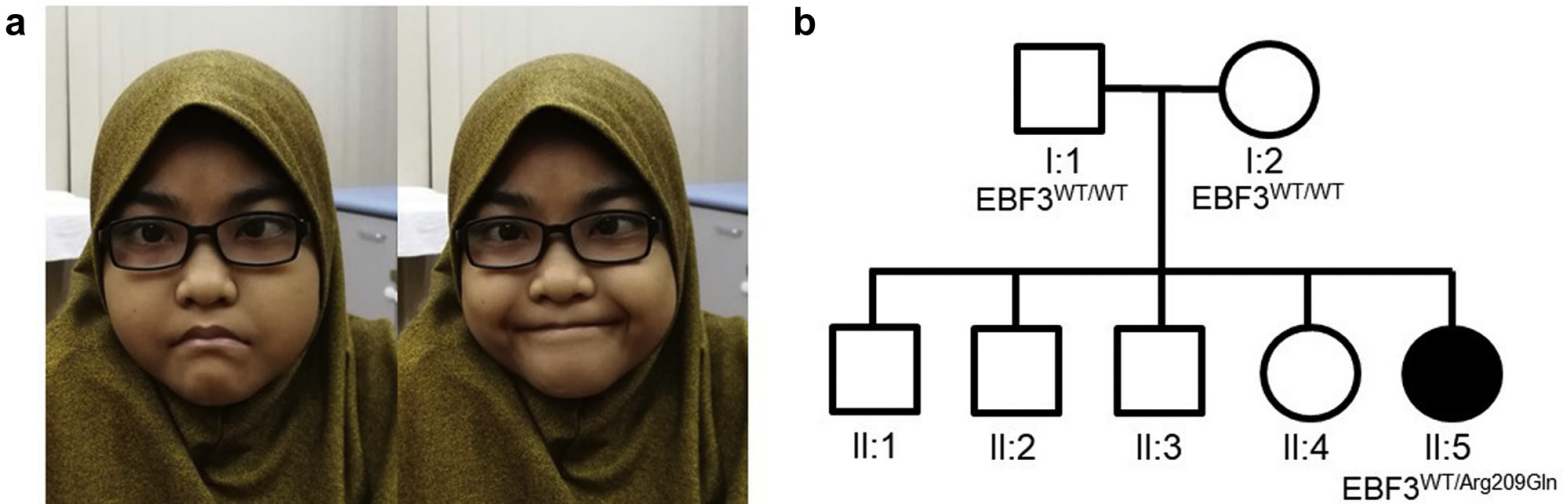
Face and family tree of the index case. (a) Proband face at rest (left frame) and the horizontal smile (right frame). (b) Pedigree showing affected proband with clinically unaffected mother, father, and elder siblings. Only the index case, II:5, carried the *EBF3* p.(Arg209Gln) variant.

A 3-year-old Malaysian girl was assigned a presumptive clinical diagnosis of UFS. Antenatal history of the proband was unremarkable but, after a normal delivery, she developed sepsis neonatally followed by recurrent urinary tract infections throughout her childhood. In the first year of life, investigations demonstrated a neurogenic bladder (in the absence of spinal cord damage) with a thickened wall, bilateral VUR, and bilateral hydroureter and hydronephrosis, the latter persisting on scans throughout childhood. When her face was at rest, the ends of her mouth were downturned and they failed to become upturned on smiling (Figure 1a), so producing an “abnormal horizontal smile” rather than the classical UFS grimace that features a downturned mouth.

The abnormal smile became less obvious as she grew up, perhaps due to improving myopathic face. She also suffered from constipation through childhood. Although the combination of renal tract disease, constipation, and abnormal smile were broadly compatible with UFS, other features were present that were atypical for UFS, including severe short stature and microcephaly; a deep philtrum, broad chin with midline cleft, and high arched palate; hypotonia with global developmental delay, being unable to walk independently and speak more than 2 words aged 3; tapering digits and fusion between the second and third toes; and a ventricular septal defect and patent ductus arteriosus that resolved spontaneously. Her brain magnetic resonance imaging scan was normal. These additional clinical features raised the possibility of a blended phenotype of UFS and another diagnosis co-occurring in the same individualS3 or of an alternative diagnosis with clinical overlap with UFS.

She died aged 17 years, after a brief illness featuring metabolic acidosis and acute kidney injury, with a plasma creatinine of 687 μM (7.77 mg/100 ml), treated with hemodialysis; blood cultures were negative and her urine showed a mixed growth of gram-negative rods.

### Genetic Analyses of the Family

Parental consent was obtained for genetic analyses. Sanger sequencing of *HPSE2* and *LRIG2* exons failed to identify relevant pathogenic variants in these genes. Whole exome sequencing of the proband was then undertaken (see Supplementary Methods and Supplementary Figure S1), and no further variants were identified in other genes (*BCN2*, *CHRM3*, the smooth muscle genes *ACTA2*, *ACTG2*, *MYH11*, *MYLK*, *MYL9*, *LMOD1,* or *MYOCD*) implicated in congenital bladder outflow obstruction.S4–S6 Instead, we identified a heterozygous missense variant c.626G>A, p.(Arg209Gln) in *EBF3* predicted to cause a missense change in a DNA-binding domain of the encoded transcription factor protein. This variant had been reported previously in an individual with HADDS,^5^, ^6^, ^7^ and it was absent from gnomAD control databases.S7 Sanger sequencing of parental DNA confirmed variant c.626G>A was *de novo*, being absent in the clinically unaffected nonconsanguineous parents (Figure 1b). The proband’s 4 siblings were also assessed as clinically unaffected, and so they were not tested for the variant. Of 8 *in silico* prediction tools used to consider the possible pathogenicity of p.(Arg209Gln), all but 1 predicted that the variant was deleterious (Supplementary Table S1).

### Sequencing *EBF3* Exons in a Cohort With Primary Nonsyndromic VUR

A previous linkage study using patient cohorts from the United Kingdom, the Republic of Ireland, and Slovenia indicated a susceptibility locus for familial primary nonsyndromic VUR on chromosome 10q26.S8 Given that *EBF3* is located at the edge of the 9 Mb region, we hypothesized that variants in *EBF3* may be enriched in such cases. Accordingly, we undertook Sanger sequencing of *EBF3* exons in 80 individuals randomly selected from the UK cohortS2 but did not identify any predicted deleterious variants (Supplementary Table S2).

## Discussion

Heterozygous variants in *EBF3* result in the rare developmental disorder, HADDS.S9 The clinical features vary among individuals, and a subset of cases has urinary tract defects and facial features, including downturned corners of the mouth. Of the 30 cases with pathogenic *EBF3* variants recorded in the literature,^8^^,^^9^^,^S1^,^S9–S11 10 (33%) were reported to have structural or functional urinary tract defects, including neurogenic bladder and VUR (Table 1). Given that VUR can be asymptomatic, and that it was not evident that all individuals with variants in *EBF3* reported in the literature had undergone urinary tract investigations, this proportion may be an underestimate. Indeed, the proportion rises to 40% when cases with recurrent urinary tract infections are included (Table 1), and whether these additional cases have functional or structural defects of the urinary tract requires further investigation. Of these individuals with *EBF3* variants and urinary tract disease, a subset had facial features (Table 1) that we believe, as in the current case, could have led to confusion with UFS. On the other hand, UFS disease is confined to the urinary tract and the face, whereas *EBF3* variants resulting in HADDS can feature developmental delay and more widespread dysmorphology (Table 2), as evidenced by the current proband.

**Table 1:** Pathogenic *EBF3* variants associated with the renal tract and/or smiling, as reported in this study and in the published literature

*EBF3* coding variant	EBF3 protein variant	Renal tract anomaly	Facial features relevant to smiling	Reference
c.280_283del	p.(Glu94Lysfs∗37)	Neurogenic bladder, VUR, recurrent UTIs	Not recorded	Sleven *et al*.S1
c.469_477dup	p.(His157_Ile159dup)	Recurrent UTIs	Not recorded	Harms *et al*.^9^
c.471C>A	p.(His157Gln)	Neurogenic bladder, VUR	Not recorded	Tanaka *et al*.^8^
c.487C>T	p.(Arg163Trp)	Atonic bladder, urethral stricture, bilateral VUR and hydronephrosis, recurrent UTIs	Downturned corners of the mouth	Blackburn *et al*.S9
c.488G>A	p.(Arg163Gln)	Impaired bladder control	Triangular-shaped facies	Chao *et al*.S10
c.488G>T	p.(Arg163Leu)	Incomplete bladder emptying, VUR	Triangular-shaped facies	Chao *et al*.S10
c.512G>A	p.(Gly171Asp)	Neurogenic bladder, bilateral VUR	Facial asymmetry	Harms *et al*.^9^
c.554+1G>T	-	VUR, recurrent UTIs, renal dysplasia	Normal	Sleven *et al*.S1
c.579G>T	p.(Lys193Asn)	VUR	Downturned corners of the mouth, minimal facial expression	Sleven *et al*.S1
c.616C>T	p.(Arg206∗)	Hydronephrosis, recurrent UTIs	Not recorded	Tanaka *et al*.^8^
c.626G>A	p.(Arg209Gln)	Normal	Lack of social smile	Tanaka *et al*.^8^
c.626G>A	p.(Arg209Gln)	Neurogenic bladder with bilateral VUR and hydronephrosis, recurrent UTIs	Abnormal horizontal smile	Proband in the current study
c.1402_1414del13	p.(Thr464Profs∗10)	Recurrent UTIs	Not recorded	Tanaka *et al*.^8^

UTI, urinary tract infection; VUR, vesicoureteric reflux.

Each row represents a single nonrelated case. The renal tract features are as described in the original publications. In addition, facial features relevant to smiling are indicated.

**Table 2:** Key teaching points

1. When an individual has vesicoureteric reflux (VUR) and/or dysfunctional urinary voiding plus an abnormal smile, clinicians should consider urofacial syndrome (UFS) and seek biallelic variants in *HPSE2* or *LRIG2.*
2. When a patient has an apparent UFS phenotype plus features such as developmental delay and broader dysmorphology, clinicians should consider hypotonia, ataxia, and developmental delay syndrome (HADDS) syndrome and seek variants in *EBF3*.
3. UFS is an autosomal recessive disease, in contrast to HADDS, which is autosomal dominant.

**Figure 2:**
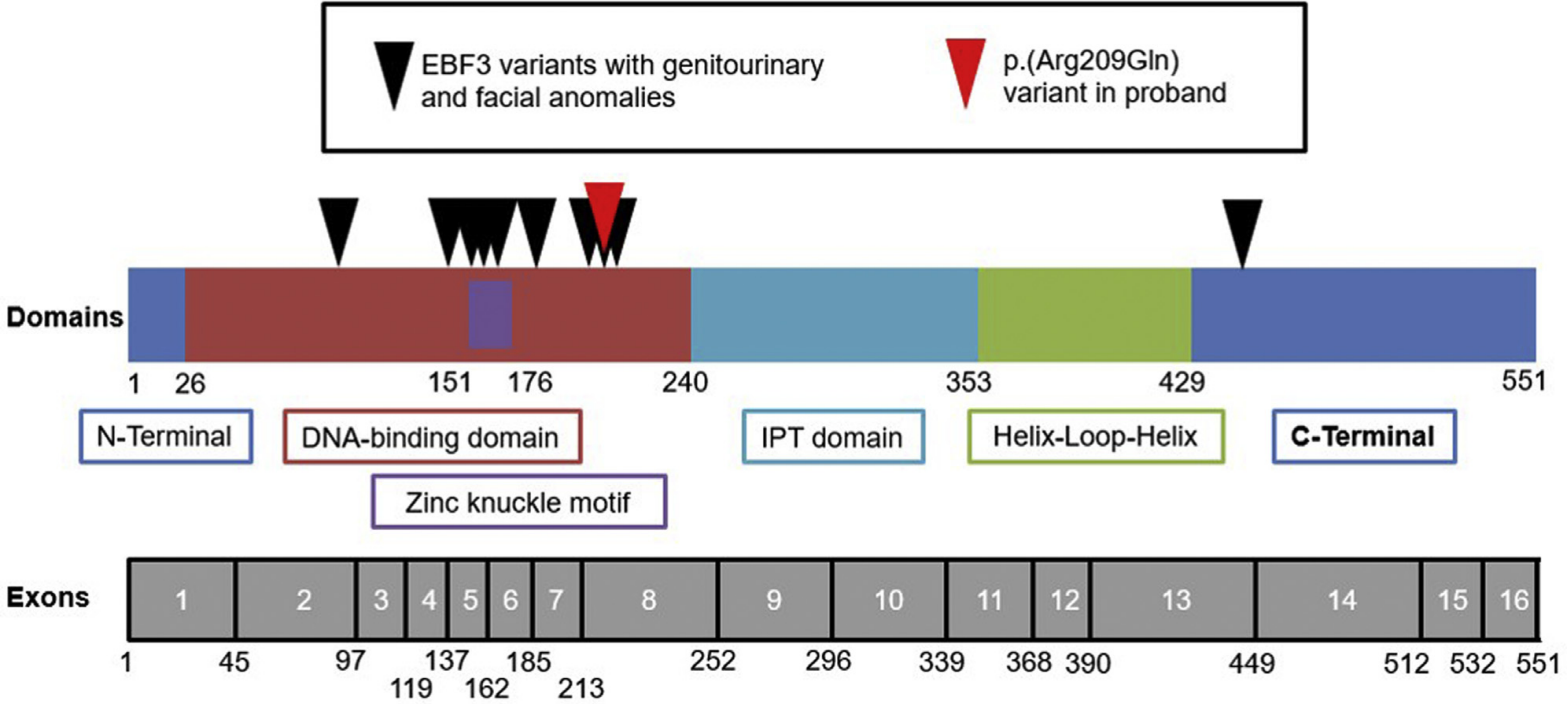
Domains and exons of the transcription factor protein encoded by *EBF3*. Numbers correspond to amino acids. Red arrowhead indicates the variant in the current proband. Black arrowheads point to positions of published variants associated with renal tract and facial anomalies, as detailed in Table 1. Zinc knuckle motif is contained within the DNA-binding domain IPT (Ig-like/plexins/transcription factor domain).

All but one of the known pathogenic variants in *EBF3* associated with urinary tract disease, fall within the DNA-binding domain of *EBF3*, as does the variant identified in our proband (Figure 2). Variants in the DNA-binding domain, and more specifically the zinc knuckle motif, have previously been associated with a reduction in EBF3 binding affinity for DNA,^9^ and are therefore likely to alter downstream gene expression, compromising normal development.

As outlined in the introduction, *HPSE2* and *LRIG2*, the genes altered in UFS, are expressed in bladder autonomic nerves, and pathogenic variants in mutant mice lead to abnormal patterns of nerves in the bladder outflow tract and the organ’s body.^5^, ^6^, ^7^ Thus, UFS features a bladder peripheral neuropathy that correlates with the functional outflow obstruction in this disease. Future studies are required to understand how *EBF3* affects the biology of the urinary tract. We note, however, that in the GUDMAP gene expression database,S12^,^S13 *Ebf3* transcripts are detected in the embryonic mouse bladder and urethral region, and in the urorectal septum of the nascent organ (https://www.gudmap.org/id/Q-48NJ@2T7-SRMW-5H12). It is currently unclear whether they are expressed in particular tissue compartments such as the nerves, muscle, or urothelium.

Aristaless-Related Homeobox (ARX) is an upstream translational repressor of *EBF3*,S10^,^S14^,^S15 and *ARX* variants have been linked to a wide variety of developmental disorders, including epileptic encephalopathy (MIM 308350), Partington X-linked mental retardation syndrome (MIM 309510), and Proud syndrome (MIM 300004).^5^, ^6^, ^7^^,^S16^,^S17 There is overlap in the range of phenotypes of individuals with *ARX* variants and those with *EBF3* variants. We speculate that the *ARX*-*EBF3* interaction may lie upstream of *HPSE2* and *LRIG2*, which are directly implicated in the pathobiology of UFS. Perhaps yet other genes associated with UFS-like disease lie downstream of the ARX-EBF3 pathway, likely with a restricted pattern of expression within key neurological pathways involved in innervation of bladder and facial muscle.

As recently reviewed,S4 the several decades search for genes implicated in primary nonsyndromic VUR, a common condition affecting at least 1% of infants, has yet to yield definitive answers, apart from *TNXB1* in a small subset of familial cases.S18 The condition is most likely genetically heterogeneous, but parametric analyses of 460 affected families comprising 1062 affected individuals showed linkage to rs7907300 located at chromosome 10q24.S8 *EBF3* is 1 of 69 genes that fall within the wider 9 Mb region surrounding rs7907300, making *EBF3* a plausible candidate for conferring VUR susceptibility. Nevertheless, we failed to detect likely pathogenic variants in *EBF3* in 80 index cases from the UK, themselves comprising approximately half of the total group analyzed in the 10q24 linkage study. Previously, we had analyzed the UK cohort by sequencing *HPSE2*^5^, ^6^, ^7^ and *LRIG2*,^5^, ^6^, ^7^ with similar negative results. However, it should be noted that patients with VUR associated with neuropathic-type bladders were excluded from this particular collection.S2 In contrast, we have identified potentially pathogenic biallelic *LRIG2* variants in 3 individuals with both severe bladder voiding dysfunction and renal failure.^5^, ^6^, ^7^^,^^5^, ^6^, ^7^ Thus, we suggest that the latter population may be enriched for individuals with Mendelian genetic causes of their urinary tract disease.

## Conclusion

The association of urinary tract disease, especially a neuropathic bladder and VUR, with an abnormal smile should alert clinicians to the possibility of not only UFS, but also HADDS (Table 2). These insights have implications for genetic counseling given that UFS is autosomal recessive, whereas HADDS is a dominantly inherited disease. *HPSE2* and *LRIG2*, the genes implicated in UFS, pattern bladder nerves. The mechanism whereby *EBF3* affects urinary tract biology requires further study (Table 2).

## Disclosure

All the authors declared no competing interests.
