# Supplementary material for: Early B-cell Factor 3–Related Genetic Disease Can Mimic Urofacial Syndrome
Source: Kidney Int Rep. 2020 Jul 14;5(10):1823–7. doi: 10.1016/j.ekir.2020.07.001 (PMC7569699; doi:10.1016/j.ekir.2020.07.001)
Supplement: Supplementary File (PDF) [file mmc1.pdf]

Supplementary Material

***Early B-cell factor 3*-related genetic disease can mimic urofacial syndrome**

J. Robert Harkness, Glenda M. Beaman et al

Supplementary Methods

The parents of the proband provided consent for familial genetic testing and for identifiable photographs to be used in this report. DNA from index cases with familial primary non-syndromic VUR were sourced from the UK VUR DNA Bank.[S2] DNA from the proband was sent to the Beijing Genomics Institution (BGI) in China where whole exome sequencing (WES) sequencing was performed using a BGI exome kit, version 4 (59M) 6G BGI-Seq500. Exome read data were aligned to the genomic reference sequence (hg19) by our in-house bioinformatics team (Genetic Medicine, St Mary's Hospital, Manchester, UK). Analysis and annotation of genome data was performed in-house using VarSeq™ v2.2 (Golden Helix, Inc., Bozeman, MT). During analysis, variants were filtered as depicted in Figure S1. For Sanger sequencing PCR primers were all designed and optimised in house. Reactions were carried out using the Veriti 96-Well Thermal Cycler (Applied Biosystems) and PCR products were purified and sequenced using an automated Applied Biosystems 3730 XL DNA Analyzer. Results were analysed using the Staden Software package. Sequencing files for patient, parent and control DNA were added to databases created using PreGap4 software, with control DNA processed as the reference sequence. Visualisation of aligned traces allowed for comparative analysis between patient and control or patient and parent sequences. Eight *in silico* prediction tools were used to interrogate variant pathogenicity: Polyphen-2 (http://genetics.bwh.harvard.edu/pph2/); Mutation taster (http://www.mutationtaster.org/); MutationAssessor (http://mutationassessor.org/r3/); SIFT (Sorting Intolerant From Tolerant) (http://sift.bii.a-star.edu.sg/); FATHMM (Functional Analysis Through Hidden Markov Models) (http://fathmm.biocompute.org.uk/inherited.html); FATHMM-MKL (Math Kernel Library) (http://fathmm.biocompute.org.uk/fathmmMKL.htm); CADD (GRCh37-v1.4)[S19]; and REVEL.[S20]

Supplementary Tables

Table S1. *In silico* tools interrogating the possible pathogenicity of the *EBF3* variant c.626G>A found in the index case.

***In silico* tool**	**Prediction**
SIFT	Damaging
PolyPhen2	Probably damaging
Mutation Taster	Disease Causing
Mutation Assessor	Predicted functional
FATHMM	Tolerated
FATHMM-MKL	Damaging
CADD	35
REVEL	0.724

Table S2. Variants detected in *EBF3* sequencing of 80 index cases of the UK VUR cohort. RSID is the reference SNP cluster ID. None of the variants are predicted to be pathogenic using *in silico* tools.

**Index cases (n)**	**cDNA and *protein* coding variants**	***EBF3* Exon or intron**	**RSID**	**gnomAD allele count/number**	**gnomAD allele Frequency**
5	c.229A>C<br>*p.(Arg77Arg)*	Exon 2	rs75074888	16766/265108	0.0632
1	c.639T>G<br>*p.(Val213Val)*	Exon 8	rs141973685	149/234652	0.000635
1	c.134+40_134+45dup	Intron 1	rs767692640	215/55380	0.003882

Supplementary Figures

Figure S1. Filtering strategy used for whole exome sequencing data analysis. Resulting variants were considered based on their association with clinical data.

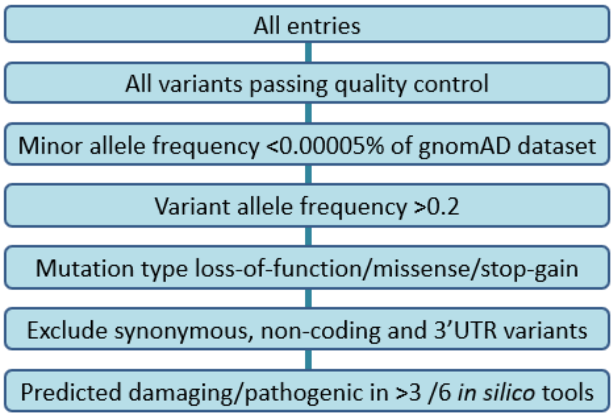

Supplementary references

S1. Sleven H, Welsh SJ, Yu J, et al. De Novo Mutations in EBF3 Cause a Neurodevelopmental Syndrome. *Am J Hum Genet*. 2017;100(1):138-150. doi:10.1016/j.ajhg.2016.11.020

S2. Lambert HJ, Stewart A, Gullett AM, et al. Primary, nonsyndromic vesicoureteric reflux and nephropathy in sibling pairs: a United Kingdom cohort for a DNA bank. *Clin J Am Soc Nephrol*. 2011;6(4):760-766. doi:10.2215/CJN.04580510

S3. Fadda A, Butt F, Tomei S, et al. Two hits in one: whole genome sequencing unveils LIG4 syndrome and urofacial syndrome in a case report of a child with complex phenotype. *BMC Med Genet*. 2016;17(1):84. doi:10.1186/s12881-016-0346-7

S4. Woolf AS, Lopes FM, Ranjzad P, et al. Congenital Disorders of the Human Urinary Tract: Recent Insights From Genetic and Molecular Studies. *Front Pediatr*. 2019;7. doi:10.3389/fped.2019.00136

S5. Kolvenbach CM, Dworschak GC, Frese S, et al. Rare Variants in BNC2 Are Implicated in Autosomal-Dominant Congenital Lower Urinary-Tract Obstruction. *Am J Hum Genet*. 2019;104(5):994-1006. doi:10.1016/j.ajhg.2019.03.023

S6. Houweling AC, Beaman GM, Postma AV, et al. Loss-of-function variants in myocardin cause congenital megabladder in humans and mice. *J Clin Invest*. 129(12):5374-5380. doi:10.1172/JCI128545

S7. Karczewski KJ, Francioli LC, Tiao G, et al. Variation across 141,456 human exomes and genomes reveals the spectrum of loss-of-function intolerance across human protein-coding genes. *bioRxiv*. August 2019:531210. doi:10.1101/531210

S8. Darlow JM, Darlay R, Dobson MG, et al. Genome-wide linkage and association study implicates the 10q26 region as a major genetic contributor to primary nonsyndromic vesicoureteric reflux. *Sci Rep*. 2017;7(1):14595. doi:10.1038/s41598-017-15062-9

S9. Blackburn PR, Barnett SS, Zimmermann MT, et al. Novel de novo variant in EBF3 is likely to impact DNA binding in a patient with a neurodevelopmental disorder and expanded phenotypes: patient report, in silico functional assessment, and review of published cases. *Cold Spring Harb Mol Case Stud*. 2017;3(3). doi:10.1101/mcs.a001743

S10. Chao H-T, Davids M, Burke E, et al. A Syndromic Neurodevelopmental Disorder Caused by De Novo Variants in EBF3. *Am J Hum Genet*. 2017;100(1):128-137. doi:10.1016/j.ajhg.2016.11.018

S11. Lopes F, Soares G, Gonçalves-Rocha M, et al. Whole Gene Deletion of EBF3 Supporting Haploinsufficiency of This Gene as a Mechanism of Neurodevelopmental Disease. *Front Genet*. 2017;8. doi:10.3389/fgene.2017.00143

S12. Harding SD, Armit C, Armstrong J, et al. The GUDMAP database--an online resource for genitourinary research. *Development*. 2011;138(13):2845-2853. doi:10.1242/dev.063594

S13. McMahon AP, Aronow BJ, Davidson DR, et al. GUDMAP: the genitourinary developmental molecular anatomy project. *J Am Soc Nephrol*. 2008;19(4):667-671. doi:10.1681/ASN.2007101078

S14. Fulp CT, Cho G, Marsh ED, et al. Identification of Arx transcriptional targets in the developing basal forebrain. *Hum Mol Genet*. 2008;17(23):3740-3760. doi:10.1093/hmg/ddn271

S15. Colasante G, Sessa A, Crispi S, et al. Arx acts as a regional key selector gene in the ventral telencephalon mainly through its transcriptional repression activity. *Dev Biol*. 2009;334(1):59-71. doi:10.1016/j.ydbio.2009.07.014

S16. Shoubridge C, Fullston T, Gécz J. ARX spectrum disorders: making inroads into the molecular pathology. *Hum Mutat*. 2010;31(8):889-900. doi:10.1002/humu.21288

S17. Olivetti PR, Noebels JL. Interneuron, interrupted: molecular pathogenesis of ARX mutations and X-linked infantile spasms. *Curr Opin Neurobiol*. 2012;22(5):859-865. doi:10.1016/j.conb.2012.04.006

S18. Gbadegesin RA, Brophy PD, Adeyemo A, et al. TNXB mutations can cause vesicoureteral reflux. *J Am Soc Nephrol*. 2013;24(8):1313-1322. doi:10.1681/ASN.2012121148

S19. Rentzsch P, Witten D, Cooper GM, et al. CADD: predicting the deleteriousness of variants throughout the human genome. *Nucleic Acids Res*. 2019;47(D1):D886-D894. doi:10.1093/nar/gky1016

S20. Ioannidis NM, Rothstein JH, Pejaver V, et al. REVEL: An Ensemble Method for Predicting the Pathogenicity of Rare Missense Variants. *Am J Hum Genet*. 2016;99(4):877-885. doi:10.1016/j.ajhg.2016.08.016
